# Investigating the Role of FlhF Identifies Novel Interactions With Genes Involved in Flagellar Synthesis in *Campylobacter jejuni*

**DOI:** 10.3389/fmicb.2020.00460

**Published:** 2020-03-24

**Authors:** Xiaofei Li, Fangzhe Ren, Guoqiang Cai, Pingyu Huang, Qinwen Chai, Ozan Gundogdu, Xinan Jiao, Jinlin Huang

**Affiliations:** ^1^Jiangsu Key Laboratory of Zoonosis, Jiangsu Co-Innovation Center for Prevention and Control of Important Animal Infectious Diseases and Zoonoses, Yangzhou University, Yangzhou, China; ^2^Key Laboratory of Prevention and Control of Biological Hazard Factors (Animal Origin) for Agrifood Safety and Quality, Ministry of Agriculture of China, Yangzhou, China; ^3^Department of Infection Biology, Faculty of Infectious and Tropical Diseases, London School of Hygiene and Tropical Medicine, London, United Kingdom; ^4^Joint International Research Laboratory of Agriculture and Agri-product Safety, Ministry of Education of China, Yangzhou, China

**Keywords:** *Campylobacter jejuni*, FlhF, transcriptional regulator, flagellar biosynthesis, pathogenesis

## Abstract

FlhF is a key protein required for complete flagellar synthesis, and its deletion results in the complete absence of a flagella and thus motility in *Campylobacter jejuni*. However, the specific mechanism still remains unknown. In this study, RNA-Seq, EMSAs, ChIP-qPCR and β-Galactosidase assays were performed to elucidate the novel interactions between FlhF and genes involved in flagellar synthesis. Results showed that FlhF has an overall influence on the transcription of flagellar genes with an *flhF* mutant displaying down-regulation of most flagellar related genes. FlhF can directly bind to the *flgI* promoter to regulate its expression, which has significant expression change in an *flhF* mutant. The possible binding site of FlhF to the *flgI* promoter was explored by continuously narrowing the *flgI* promoter region and performing further point mutations. Meanwhile, FlhF can directly bind to the promoters of *rpoD*, *flgS*, and *fliA* encoding early flagellin regulators, thereby directly or indirectly regulating the synthesis of class I, II, and III flagellar genes, respectively. Collectively, this study demonstrates that FlhF may directly regulate the transcription of flagellar genes by binding to their promoters as a transcriptional regulator, which will be helpful in understanding the mechanism of FlhF in flagellar biosynthetic and bacterial flagellation in general.

## Introduction

*Campylobacter jejuni* flagella are considered the main virulence factor playing a key role in many important biological activities, such as motility, chemotaxis, adhesion, secreting virulence and colonization factors (Beeby, 2015; Burnham and Hendrixson, 2018; Subramanian and Kearns, 2019). *C. jejuni* is a microaerophilic, Gram-negative bacterium, and is the leading cause of foodborne related gastroenteritis worldwide (Flint et al., 2016; Burnham and Hendrixson, 2018). It generates a single unsheathed flagellum at one or both poles of the cell (Hendrixson and Dirita, 2003; Matsunami et al., 2016; Liang and Connerton, 2018). Flagellar biosynthesis is complicated requiring expression of more than 50 genes highly regulated by a complex regulatory network that ensures the coordinate expression to construct an intact flagella organelle (Balaban et al., 2009; Grinnage-Pulley et al., 2016; Liang and Connerton, 2018). Given the importance of flagella, a thorough understanding of its assembly is necessary.

In many bacteria, flagella genes are grouped together into operons and are controlled by global regulatory factors (Chilcott and Hughes, 2000; Prouty et al., 2001; Dasgupta et al., 2003; Liu and Ochman, 2007). In *C. jejuni* however, scattered flagellar genes lack a global regulatory factor, such as FlhDC in *E. coli*, creating a challenge for exploring the regulation mechanism on the flagellar synthesis. The formation of flagella is divided into three cascades (Balaban et al., 2009). In the early stage, σ70 factor-dependent class I genes are synthesized, including flagellar export apparatus (FEA, consisting of FlhA, FlhB, FliF, FliO, FliP, FliQ, and FliR), σ28, σ54 factors and FlgSR TCS. Then class II genes and class III genes are synthesized in sequence (Wösten et al., 2004; Joslin and Hendrixson, 2009; Lertsethtakarn et al., 2011). Although flagella have long been extensively studied, there remains a gap in our knowledge as to the regulation mechanisms of flagellar proteins synthesis (Gao et al., 2014).

One identified protein that primarily affects flagellar biosynthesis is FlhF. In other species, the disruption of *flhF* can lead to a range of different phenotypes, including reduced flagellar gene expression, decreased or absent motility, decreased virulence, abnormal flagella assembly and number, and even no flagellation (Kazmierczak and Hendrixson, 2013; Burnham and Hendrixson, 2018). In *C. jejuni*, an *flhF* mutant leads to a complete loss of motility and a non-flagellar phenotype. Despite FlhF having a crucial influence on the flagellar synthesis, the specific genetic regulatory mechanisms are unclear (Kim et al., 2012; Kazmierczak and Hendrixson, 2013; Schuhmacher et al., 2015). Thus far, many studies of FlhF have focused on its role in determining the position and number of flagella substructure. FlhF is a member of the signal recognition particle (SRP) associated GTPase family, however the exact function is not well defined (Kim et al., 2012; Guttenplan et al., 2013; Schniederberend et al., 2013; Schuhmacher et al., 2015; Gulbronson et al., 2016). Other studies have identified the influence on flagellar gene expression, nevertheless, the results reported have not always been in alignment (Niehus et al., 2004; Correa et al., 2005; Murray and Kazmierczak, 2006; Balaban et al., 2009; Kim et al., 2012). In *C. jejuni*, the specific mechanisms of FlhF still need to be explored in depth.

Transcriptional regulators are important for biological response and their adaptability to different conditions in the organism (Galán-Vásquez et al., 2016). Some organisms have many transcriptional regulators, for example *E. coli* has seven σ factors, *Bacillus subtilis* has 19, *Streptomyces coelicolor* over 60 and more than 100 in *Sorangium cellulosum* (Bervoets and Charlier, 2019). However, the *C. jejuni* genome carries only three sigma factors, RpoD, RpoN, and FliA (Hwang et al., 2011). Meanwhile, there are approximately 34 other transcription factors in *C. jejuni* (Wösten et al., 2004; Nachamkin et al., 2008; Grinnage-Pulley et al., 2016). Genome-wide analysis indicates that *C. jejuni* strains contain between approximately 1,650 and 1,800 genes (Parkhill et al., 2000; Hofreuter et al., 2006; Parker et al., 2006). So this indicates that all *C. jejuni* biological functions, including bacterial replication, adaptation to environments and bacterial pathogenicity are largely controlled by a limited number (∼2% of the total) of *C. jejuni* proteins (Nachamkin et al., 2008). Therefore, the discovery of new transcriptional factors and transcriptional regulation mechanisms is essential to better analyze *C. jejuni* biology.

Considering the crucial influence of FlhF on the flagellar synthesis, we speculate that FlhF may directly regulate flagellar genes expression like a transcription factor. We have applied EMSA and ChIP-qPCR to explore the transcriptional function of FlhF here. The overall influence of FlhF on flagellar gene expression was analyzed by RNA-Seq. We further explored whether FlhF directly regulates early flagellar regulatory factors including RpoD, RpoN, FliA, FlgSR two-component system (TCS) (Petersen et al., 2003; Wösten et al., 2004). Collectively, our results firstly reveal that FlhF may directly regulate flagellar genes transcription by binding the promoters of specific genes (Huffman and Brennan, 2002). Moreover, we proposed a pattern for the feasible transcriptional regulatory pathways of FlhF in flagellar synthesis which will be helpful in understanding the *C. jejuni* flagella biosynthetic pathway and bacterial flagellation in general.

## Materials and Methods

### Bacterial Strains and Plasmids

All strains and plasmids used in this study are listed in Supplementary Table S1. Briefly, *C. jejuni* 81–176 strain and its derivatives were typically grown on *Campylobacter* blood-free selective agar containing charcoal cefoperazone deoxycholate (CCDA) (Oxoid, Basingstoke, United Kingdom) at 42°C under microaerobic conditions (85% N_2_, 10% CO_2_, and5% O_2_). *Escherichia coli* DH5α and BL21 strains were grown at 37°C in Luria-Bertani (LB) broth or on LB agar (Ren et al., 2018). As required, antibiotics were added to the medium for *C. jejuni* or *E. coli* at the following concentrations: 100 μg ml^–1^ ampicillin, 50 μg ml^–1^ kanamycin, or 20 μg ml^–1^ chloramphenicol. The plasmid pMD-19T (simple) (TaKaRa, Dalian, China) was used as a suicide vector in cloning and strain construction. Plasmid pRK2013 (Biomedal, Seville, Spain) is a helper plasmid for triparental mating conjugation, while pUOA18 is a *C. jejuni* shuttle vector courtesy of Qijing Zhang (Iowa State University, Ames, United States).

### Construction of *C. jejuni* Mutant and Complemented Strains

To inactivate *flhF*, its flanking regions and the Kan^R^ cassette were amplified from *C. jejuni* genome and pRY107, then, ligated into pMD-19T (simple) using T4 ligase to obtain a suicide plasmid. The primers used for strain construction are listed in Supplementary Table S2. The suicide plasmid was electroporated into *C. jejuni* competent cells, and the resulting transformants were selected on CCDA agar containing 50 μg ml^–1^ kanamycin. The *flhF* complement strain was constructed by the shuttle vector pUOA18 as previously described (Ren et al., 2018). The target gene was amplified and ligated directly downstream of the promoter P*metk* in the shuttle vector. The recombinant plasmid was mobilized into the *flhF* mutant strain by triparental mating using *E. coli* DH5α transformant containing pUOA18-P*metk-flhF* plasmid as the donor strain and DH5α (pRK2013) as the helper strain, by the method described by Miller et al. (2000). The cultures of *flhF* mutant strain were removed and resuspended in PBS to an OD_600_ of 1.0. Overnight cultures of the donor and helper *E. coli* strains were subcultured into Luria-Bertani (LB) broth and grown to an OD_600_ of 1.2. Cells were mixed at a ratio of 1:1:10 (donor/helper/recipient), spotted onto the Mueller-Hinton (MH) agar plate (BD, United States), and incubated overnight at 42°C under microaerophilic conditions. The mating spot was then resuspended in Mueller-Hinton (MH) broth and plated onto CCDA plate amended with Polymyxin B (6.7 μg/ml), Rifampicin (10 μg/ml), Trimethoprim (5 μg/ml), and chloramphenicol (20 μg/ml). The plates were examined after 3–5 days for the appearance of *C. jejuni* colonies, and verified by polymerase chain reaction (PCR).

### Expression and Purification of FlhF-His_6_, CmeR-His_6_

FlhF and CmeR proteins were expressed in *E. coli* DE3 system containing pET-30-FlhF and pCold I-CmeR, respectively. The *flhF*,*cmeR* genes were amplified from *C. jejuni* genome, then, ligated into pET-30a (between *Bam*HI and *Xho*I sites) and pCold I (between *Xho*I and *Sal*I sites), respectively, using the ClonExpress II one-step cloning kit (Vazyme, Nanjing, China) to generate the recombinant expressing plasmids used to transform *E. coli* DE3. Then they were cultured on a LB plate containing 50 μg/ml kanamycin and verified by PCR and nucleotide sequencing. The FlhF-His_6_, CmeR-His_6_ protein were expressed and purified from the soluble extract by affinity chromatography using a HiTrap Ni^2+^-chelating column. The purification procedure followed the instructions of the manufacturer of the His Bind Purification Kit (Novagen, EMO Millipore corp, Billerica, MA, United States). Purified Protein were analyzed by SDS-PAGE (Supplementary Figure S5) and stored at −70°C.

### Construction of Promoter-*lacZ* Transcriptional Fusions

The promoter regions of interest were amplified from *C. jejuni* genome and ligated into pMW10 to obtain promoter-*lacZ* transcriptional fusion plasmids (Wosten et al., 1998). With the aid of plasmid pRK2013, the transcriptional fusion plasmids were introduced into WT and the *flhF* mutant strain by amphiphilic mating conjugation, which was cultured on a CCDA agar containing 50 μg/ml kanamycin, and verified by PCR. The primers used for strain construction are listed in Supplementary Table S2.

### RNA Isolation and Quantitative Real-Time PCR

*Campylobacter jejuni* 81-176, FlhF-kan mutant strain was grown on CCDA plates and suspended in Mueller-Hinton (MH) broth (BD, United States) with an initial OD_540_ of 0.07, cultured for 8 h with 42°C, 120 rpm, and total RNA was extracted by using an RNeasy plus mini kit (Qiagen, Hilden, Germany) obeying the instructions of manufacturer. cDNA was synthesized by a total of 500 ng of RNA using RT reagent kit (TaKaRa, Dalian, China), which was subjected to quantitative real-time PCR (qRT-PCR) or stored at −70°C until use. qRT-PCR was carried out in an ABI PRISM 7500 Real-Time PCR System (Applied Biosystems, Foster City, CA, United States) using a FastStart Universal SYBR Green Master (ROX) (Roche Diagnostics, Mannheim, Germany). Cycling conditions were as follows: 2 min at 50°C, then 40 cycles of 30 s at 95°C and 34 s at 60°C. As previously described, relative genes expression was calculated using the 2^–ΔΔCT^ method (Livak and Schmittgen, 2001). All specific primers are listed in Supplementary Table S2, in which the *glyA* gene was used as an endogenous control. A series of 10-fold diluted cDNA were used as templates and the standard curves were generated for each candidate genes. The PCR efficiency (E) was calculated using the following formula (Pfaffl, 2001):

E=10)(-1/-slope

### RNA-Seq

To analyze the transcriptome, RNA-Seq libraries were generated for six bacterial samples [2 bacterial strains (WT, Δ*flhF*) × 3] from cDNA using instructions according to the TruSeq^TM^ RNA sample preparation Kit (Illumina, San Diego, CA, United States). As previously described, the quality control of the total RNA samples was performed using a 2100 Bioanalyzer (Agilent) and the ND-2000 (NanoDrop Technologies). Only high-quality RNA samples (OD260/280 = 1.8∼2.2, OD260/230 ≥ 2.0, RIN ≥ 6.5) were used to construct sequencing library (Zhang et al., 2019). The cDNA was then synthesized according to the SuperScript double-strand cDNA synthesis kit (Invitrogen, Carlsbad, CA, United States). The RNA-Seq libraries were subjected to quality inspection using an Agilent 2100 Bioanalyzer (Agilent Technologies) and sequenced on an Illumina HiSeq4000 (Illumina Inc., San Diego, CA, United States), which was biologically replicated in a separate experiment by Majorbio (Shanghai, China). Sequence reads were processed and mapped as previously described (Garber et al., 2011). Gene expression (FPKM) and differential expression levels were analyzed using Rsem^1^ and edgeR software^2^. For functional annotation of mRNA, we used Blastx with the NCBI-NR database, String, Swissprot and the Kyoto encyclopedia of genes and genomes (KEGG) database. Statistical analysis according to the method described in the previous period, *P*-value < 0.05 was considered to be statistically significant. All RNA-Seq data was uploaded to the EBI ENA databased (Accession number PRJEB34440).

### Electrophoretic Mobility Shift Assay (EMSAs)

The EMSAs were performed as follows: PCR fragments encompassing the promoters of genes with FAM-labeled were amplified using genomic DNA of *C. jejuni* 81-176 as a template. The DNA fragments were gel-purified using MiniBEST Agarose Gel DNA Extraction Kit (Takara, Japan). Each PCR product (≈5 ng) was mixed with increasing concentrations of purified FlhF-His_6_ in a final volume of 20 μl buffer containing 10 mM Tris-HCl (pH 8.0), 50 mM KCl, 1 mM DTT, 0.5 mM EDTA and 5% glycerol. The reactions were incubated for 30 min at 25°C and then loaded with 10×EMSA loading buffer on 6% polyacrylamide non-denaturing gels in 0.5 × Tris-borate-EDTA buffer. Each reaction was verified to be specific by adding 10-fold non-specific competitor [Poly(dI:dC)]. For a negative control, synthesized His-tag was incubated with *flgI* promoter, denoted as negative control (NC). For positive controls, cmeA promoter was incubated with the purified CmeR protein, and *cmeA* promoter alone, denoted as positive control (PC) (Cagliero et al., 2007).

### ChIP-qPCR

The 3 × FLAG-tagged strain (WT *flhF*-FLAG) was grown under microaerobic conditions and then pelleted by centrifugation. As described previously (Blasco et al., 2012), however, with some variation, ChIP was performed based on established methods as follows. Formaldehyde was added to bacterial cells (1% final concentration) for cross-linking and then incubated at room temperature for 25 min. Reactions were quenched with 0.5 M glycine, and samples were pelleted and washed three times with PBS. The samples were then used for ChIP following the Chromatin Immunoprecipitation kit (Millipore, United States) protocol. The antibody used was the anti-FLAG mouse monoclonal antibody (Sigma). For ChIP-qPCR experiments, untreated chromatin was de-cross-linked by boiling for 10 min and purified for use as the “input” control. The relative enrichment of candidate gene promoters was performed with qRT-PCR and represents the value of the immunoprecipitated DNA divided by the input unprecipitated DNA. These values were normalized to the values obtained for each promoter precipitated using untagged wild-type in order to account for non-specific enrichment. The results represent the mean enrichment measured via qPCR in at least three biological replicate experiments.

### β-Galactosidase Assay

*Campylobacter jejuni* cells carrying the transcriptional fusion plasmids were grown on CCDA plates and suspended in MH broth with the same OD_600_. The cells with centrifugation were suspended thoroughly with 1 ml Z buffer (60 mM Na_2_HPO_4_, 40 mM NaH_2_PO_4_, 10 mM KCl, 1 mM MgSO_4_, pH 7.0) and shaken vigorously to lyse the cells with adding 30 μl of chloroform and 0.1% SDS. The assays were performed at 37°C with 200 μl ONPG (*O*-nitrophenol-β-D-galactopyranoside, 4 mM, Sigma) and monitored at 420 nm (Cagliero et al., 2007). β-Galactosidase activities were calculated in Miller Units using the formula given below: β-Galactosidase activity = A420 × 1000 × min^–1^ × ml^–1^ × A600^–1^. The results were reported as the mean of three biological replicates.

## Results

### FlhF Has an Overall Impact on the Transcription of Flagella Components

**FIGURE 1:**
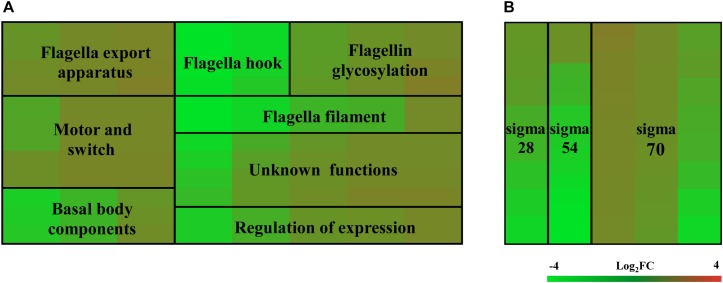
Heat map of relative mRNA expression of *C. jejuni* flagella genes. The transcription of flagella genes in the RNA-Seq were analyzed according to their function and substructure affiliation **(A)**, as well as transcription cascade **(B)**. Flagella hook genes and class II genes were significantly affected according to the ANOVA analysis at *P* < 0.05. Green and red in the heat map represent down-regulation and up-regulation of genes in the *flhF* mutant relative to the wild-type strain, with more saturated colors representing a larger differential effect as listed in the bar.

**FIGURE 2:**
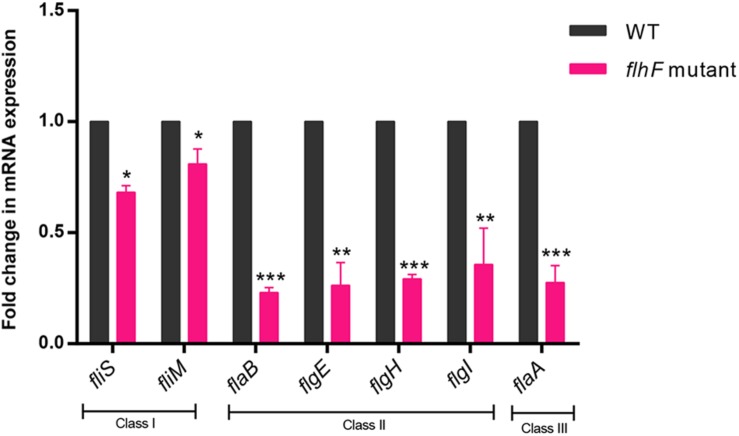
qRT-PCR for verification of RNA-Seq data. qRT-PCR data demonstrated correlation with the RNA-Seq data when investigating the transcript fold-change difference between WT strain and the *flhF* mutant strain. Each sample was examined in three biological replicates and was repeated with three technical replicates. Data are presented as mean ±SD. Data were analyzed by using a one-sample *t*-test (* *P* < 0.05, ** *P* < 0.01, *** *P* < 0.001).

To investigate which genes are regulated by FlhF on the transcriptional level, we performed high-throughput RNA sequencing based on the genetic background (*flhF* mutant vs. wild-type). RNA-Seq data (Supplementary Table S3) showed all the modulated flagellar related genes are down-regulated in the mutant, suggesting FlhF has a positive role in flagellar gene transcription. Among these down-regulated genes, 26 genes are involved in the process of flagellar biosynthesis. Grouping these genes according to their function and substructure affiliation showed a general trend of down-regulation from flagella export apparatus, motor/switch components, an unknown function (hypothetical genes with unknown function), flagellin glycosylation, transcription regulators, flagella basal body, the filament, to the most down-regulated which was the flagella hook (Figure 1A). Flagellar genes are classified into three cascades, class I genes are σ70 dependent, while class II and class III genes require σ54 and σ28 factor, respectively (Ren et al., 2018). A one-way ANOVA analysis showed that class I, III, and II genes were significantly modified when comparing the *flhF* mutant to the respective wild-type strain (Figure 1B). qRT-PCR was performed to verify the results of the RNA-Seq data. Seven flagellar genes that belong to different cascades were randomly selected (Figure 2). The amplification efficiency of each pair of primers were close to 2 (Supplementary Figure S1) and the reference gene *glyA* was constantly expressed under this experimental condition due to the relatively stable CT values (Supplementary Table S4).

### FlhF May Directly Regulate *flgI* Transcription by Binding Its Promoter

**FIGURE 3:**
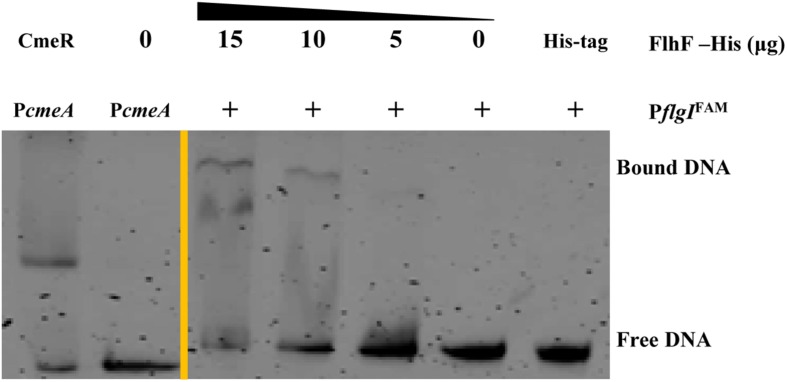
FlhF directly interact with the *flgI* promoter. EMSA demonstrated that the fluorescently labeled DNA probes of the *flgI* promoter were incubated with purified FlhF-6His at different concentrations. Each reaction was verified to be specific by adding 10-fold non-specific competitor [Poly(dI:dC)]. For a negative control, synthesized His-tag was incubated with *flgI* promoter, denoted as negative control (NC). For positive controls, *cmeA* promoter was incubated with the purified CmeR protein, and *cmeA* promoter alone, denoted as positive control (PC). The “+” symbol indicates the presence of FlhF-6His.

**FIGURE 4:**
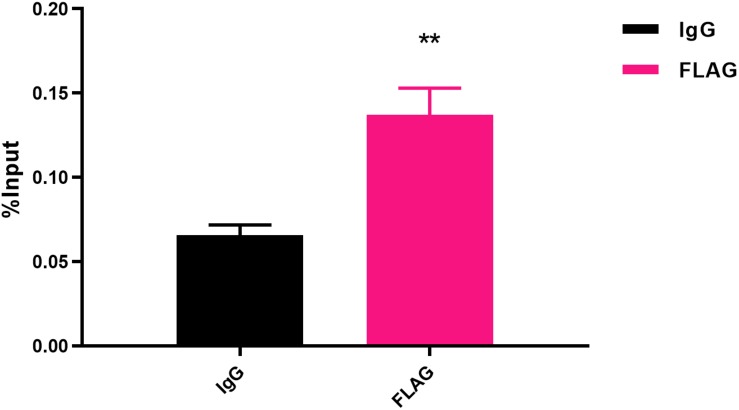
Fold enrichment of the *flgI* promoter in ChIP samples measured via ChIP-qPCR. The results show the promoter of *flgI* was extremely enriched in FlhF-ChIP samples, and the relative quantity was significantly higher than in the IgG control samples. Genome fragments isolated from wild-type *flhF*-FLAG strain were immunoprecipitated with corresponding antibodies, and analyzed by real-time PCR using primer sets corresponding to transcriptional start site regions of the *flgI*. For ChIP-qPCR experiments, the sample was cross-linked and sonicated to generate small DNA fragments, and then divided into three parts. The first was used as an input control. The second was incubated with normal rabbit IgG that will not bind to nuclear proteins to generate immuno-precipitated DNA (IgG). The third was as ChIP-FLAG immunoprecipitated sample (FLAG) (O’Geen et al., 2010). Results represent mean enrichment as measured by qPCR in at least three biological replicate experiments. Data are presented as mean ±SD. Data were analyzed by one-sample *t*-test (** *P* values are < 0.01, but > 0.001) to estimate the significance of fold change between FlhF-ChIP samples and IgG control samples.

To determine the transcriptional function of FlhF, six genes with significantly different expression were randomly selected from the RNA-Seq results, including *fliK*, *flaB*, *flgE*, *flaA*, *flgL*, *flgI*, to explore whether FlhF binds their promoters by Electrophoretic mobility shift assay (EMSA). Our results demonstrated that the purified FlhF-His_6_ bound to the promoter of *flgI* (Figure 3), the flagellar P-ring component, but did not bind to the promoters of other genes (Supplementary Figures S2A–E). The results were further verified by Chromatin Immunoprecipitation quantitative PCR (ChIP-qPCR) analysis (Figure 4). We selected *flgI* and *flaB* to perform ChIP-qPCR, which showed that the promoter of *flgI* was extremely enriched in FlhF-ChIP samples, and the relative quantity was significantly higher than in the IgG control samples (Figure 4), while the promoter of *flaB* was not enriched in the FlhF-ChIP samples (Supplementary Figure S3). In summary, all results demonstrated that FlhF may directly regulate *flgI* as a positive transcriptional regulator.

### FlhF-Binding Site in the *flgI* Promoter

**FIGURE 5:**
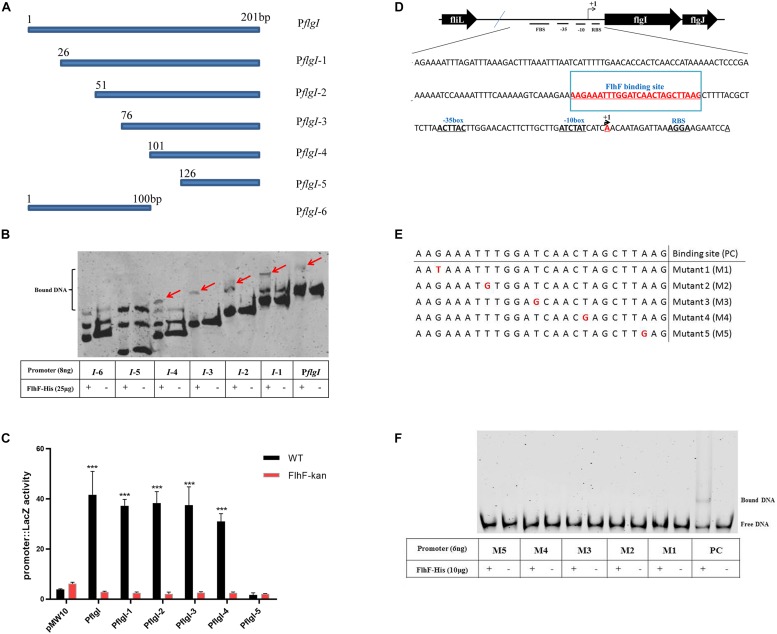
FlhF- binding site in the *flgI* promoter. **(A)** A diagram of the *flgI* promoter (a 201 bp fragment of the promoter adjacent to the start codon “ATG”) and the six regions carried on the DNA fragments used in this assay. Fragment names are displayed in figure. **(B)** EMSA analysis of FlhF specifically binding to the *flgI* promoter. All seven fragments (8 ng) with FAM labeled in **(A)** were incubated with purified FlhF-His_6_ (25 μg). The “+” and “–” symbols indicate cultures incubated with FlhF-His_6_ and without FlhF-His_6_, respectively. The red arrows represent the bound DNA. Each reaction was verified to be specific by adding 10-fold non-specific competitor [Poly(dI:dC)]. **(C)** β-Galactosidase assay for verification of EMSA results. Expression of the β-Galactosidase activities in WT and *flhF* mutant strains containing different fragments. The activity is expressed as the mean ± SE from three biological experiments. The strains containing pMW10 served as negative control. Data were analyzed by *t*-test to estimate the significance of fold change between WT and FlhF-kan samples. The symbol “***” means *P* < 0.001. **(D)** Diagram showing the promoter region of the *flgI* gene. The ribosome-binding site (RBS) are underlined and the transcription start sites are labeled as +1 and marked in red. The −35/−10 motif were located directly upstream of the transcriptional start site +1A. We speculated that the FlhF-binding site was underlined and marked in red (−76 to −51). **(E)** A diagram of five different point mutations of possible FlhF-binding site on P*flgI* (a 26 bp fragment) and the six DNA fragments used in this assay. Fragment names are displayed in figure. PC signifies positive control. **(F)** EMSA analysis of the binding of FlhF to the five different point mutations. All six fragments (6 ng) with FAM labeled in **(E)** were incubated with purified FlhF-His_6_ (10 μg). The “+” and “–” symbols indicate cultures incubated with FlhF-His_6_ and without FlhF-His_6_, respectively. Each reaction was verified to be specific by adding 10-fold non-specific competitor [Poly(dI:dC)].

To delineate the contribution of portions of the *flgI* promoter for binding of FlhF, the *flgI* promoter was divided into six fragments (Figure 5A), which were amplified with FAM-labeling and ligated into pMW10 to perform EMSA and β-Galactosidase assay here. EMSA results showed that the purified FlhF-His_6_ bound to the fragments 1–4 of the *flgI* promoter, but did not bind to the fragments 5–6 (Figure 5B), which demonstrated that the putative binding site of FlhF in the *flgI* promoter was between 101–125 bp. β-Galactosidase assays performed to verify the results from 5B and showed that the fragments 1–4 of the *flgI* promoter had notable differences between the WT and *flhF* mutant strain, but fragment 5 has no difference with negligible activity, which was consistent with the EMSAs (Figure 5C). We used strains containing pMW10 served as a negative control. Therefore, we speculated the FlhF-binding site (FBS) in *flgI* promoter is the region “−76 to −51” (AAGAAATTTGGATCAACTAGCTTAAG) (Figure 5D). To further investigate the necessity of this motif for the binding of FlhF to the *flgI* promoter (P*flgI*), we selected a point mutation every 5 bp on the possible FlhF-binding site to generate five different point mutation fragments which were amplified with FAM-labeling (Figure 5E). EMSA results showed that the purified FlhF-His_6_ bound to the 26 bp possible FlhF-binding site of the *flgI* promoter, but did not bind to the five different point mutation fragments (Figure 5F), which demonstrated that the five-point mutations abolish binding of FlhF to P*flgI*.

### FlhF Directly Regulates Flagellar Gene Regulators *rpoD*, *fliA*, *flgS*

**FIGURE 6:**
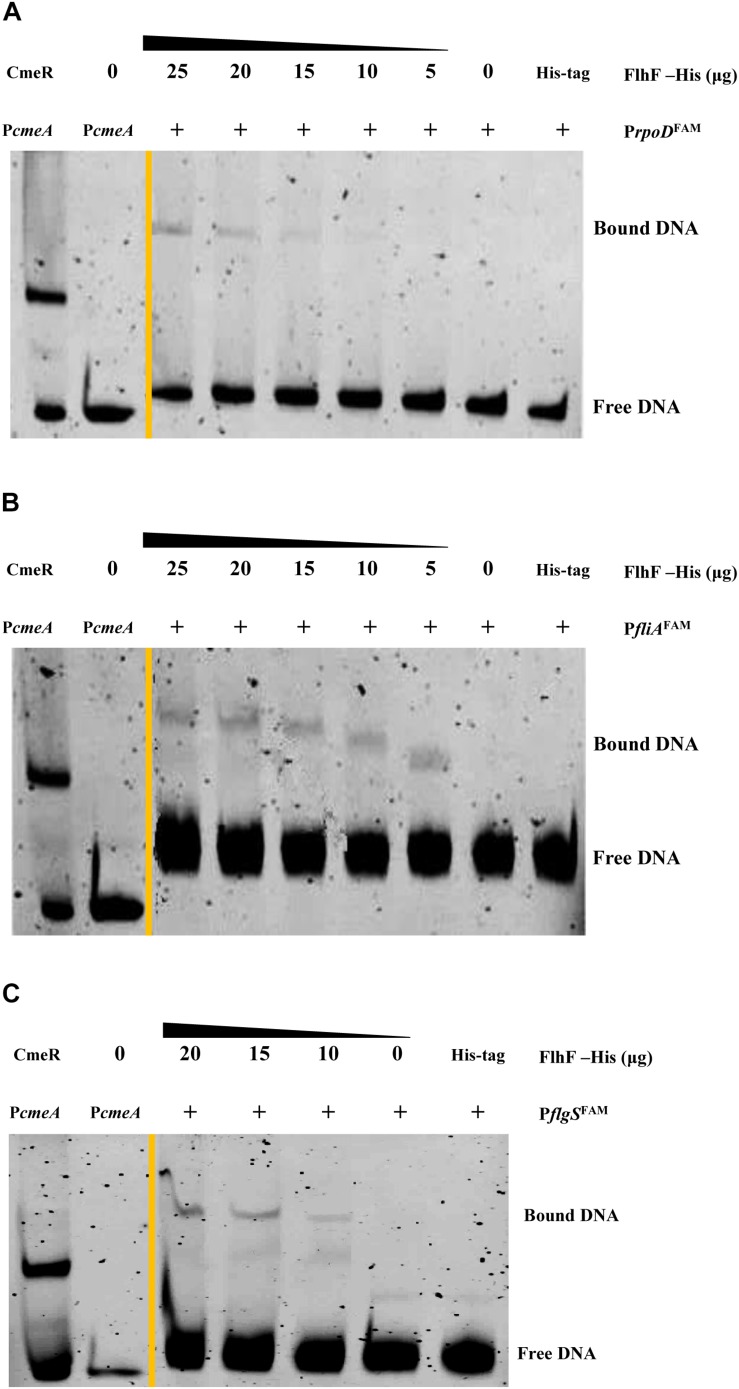
FlhF directly interact with the promoter of *rpoD, fliA, flgS*. The fluorescently labeled DNA probes of the promoter were incubated with purified FlhF-HIS_6_ at different concentrations and promoters **(A)** *rpoD*; **(B)** *fliA* and **(C)** *flgS* respectively. For a negative control, synthesized His-tag was incubated with *flgI* promoter, denoted as negative control (NC). Each reaction was verified to be specific by adding 10-fold non-specific competitor Poly(dI:dC)]. For positive controls, *cmeA* promoter was incubated with the purified CmeR protein, and *cmeA* promoter alone, denoted as positive control (PC). The “+” symbol indicates the presence of FlhF-6His.

RNA-Seq results demonstrated that FlhF has an overall impact on the transcription of flagellar components. We hypothesize that FlhF may regulate flagellar gene expression by directly regulating key regulatory factors *rpoD*, *rpoN*, *fliA*, *flgSR* TCS during flagellar synthesis. Hence, we explored whether FlhF regulates them directly by binding their promoters by EMSA. Results showed that the purified FlhF-His_6_ bound to the promoters of *rpoD*, *fliA*, *flgS* (Figures 6A–C), but did not bind to the promoters of *rpoN* and *flgR* (Supplementary Figures S4A,B).

## Discussion

In *C. jejuni*, flagella is a major virulence factor with a complex synthesis process. FlhF is one of several key proteins that influence flagellar biosynthesis. Deletion of *flhF* results in a non-motile and non-flagellar phenotype. Despite FlhF having a crucial influence on flagellar synthesis, the specific mechanism of its role remains unclear (Kim et al., 2012; Kazmierczak and Hendrixson, 2013; Schuhmacher et al., 2015). In this study, RNA-Seq was performed to investigate the role of FlhF further. Previous studies involved in functionality of FlhF influencing the flagellar genes expression are varied depending on the bacteria of choice (Niehus et al., 2004; Correa et al., 2005; Murray and Kazmierczak, 2006; Lertsethtakarn et al., 2011; Kim et al., 2012). In *Helicobacter pylori*, FlhF was found to primarily affect class II and class III flagella genes expression. For *Pseudomonas aeruginosa*, a mutation of FlhF resulted in decreased transcription of the class IV gene *fliC*. In *Vibrio cholerae* and *Vibrio vulnificus*, FlhF positively affected the transcription of class III and class IV flagella genes. However, our results revealed that FlhF may act as an activator of flagellar genes and thus an overall influence on flagellar gene expression in *C. jejuni* (Figure 2).

In order to further explore how FlhF affects flagellar expression, in our study we investigated the putative function of FlhF directly influencing flagella synthesis by binding the promoter of flagellar genes. FlhF may positively control *flgI* expression by binding to promoter of *flgI* (Figures 4–5), which is the flagellar P-ring component (Boll and Hendrixson, 2013). The P-ring together with L-ring are thought to be required for smooth rotation, functioning as a sleeve in many motile bacteria (Hizukuri et al., 2006, 2008). We found *flgI* and *flgH* had 6.6-fold and 8-fold reduction in expression respectively after the deletion of *flhF* using RNA-Seq (Supplementary Figure S3), which indirectly supported our hypothesis that *flhF* has a potential regulatory role. Additionally, the protein-binding site and DNA binding site are important for transcriptional regulators. Our results have revealed that the possible binding site of FlhF in the *flgI* promoter is “AAGAAATTTGGATCAACTAGCTTAAG” (Figure 5). Five different point mutations were generated to further investigate that the complete promoter site may be necessary for binding of FlhF to P*flgI*. Meanwhile, since FlhF mainly affects class II genes, we speculated that there may be other genes besides *flgI* that can be directly regulated by FlhF. ChIP-seq will be performed to identify further hits in the future.

**FIGURE 7:**
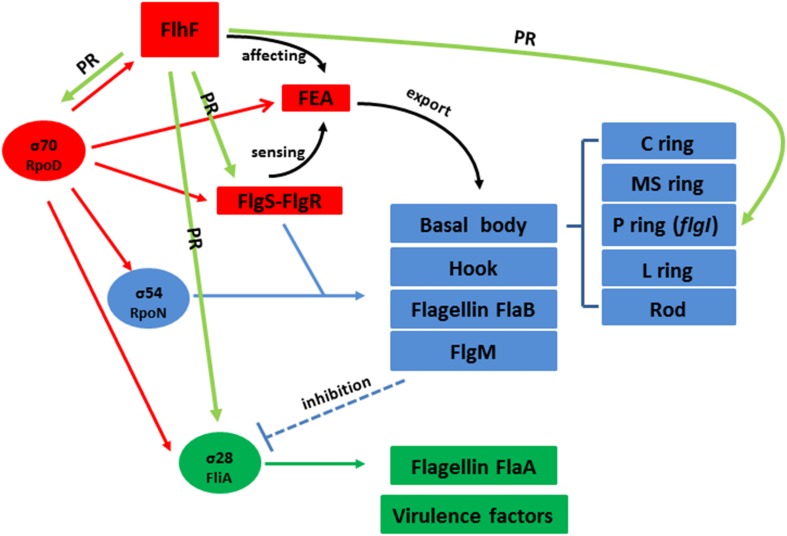
Hypothetical model for the regulatory pathway of FlhF in the *C. jejuni* flagellar system. *C. jejuni* flagellar genes are classified into three cascades, class I genes (red) are σ70 (*rpoD*) dependent, while class II (blue) and class III (green) genes require σ54 (*rpoN*) and σ28 (*fliA*) factor, respectively. The σ70 factor-dependent class I genes are constitutively expressed. FlhF acts before the flagella export apparatus (FEA) formation. The FEA formation will also be detected by the FlgSR two-component system (TCS) and activates the σ54 factor-dependent class II genes. After FlgM is secreted from the cell, the inhibition of the σ28 factor-dependent class III genes is relieved and they are expressed. In our study, we speculate that FlhF may directly regulate the synthesis of class I and III flagellar genes by binding to the *rpoD* and *fliA* promoters, activating σ70 and σ28 factor respectively. For the class II flagellar genes, FlhF may bind P*flgS* to stimulate *flgS* phosphorylation of *flgR*, cooperating with σ54 factor, indirectly to initiate class II gene transcription synthesis. In addition FlhF may also directly regulate the specific class II gene *flgI* by binding to its promoter. “PR” signifies “positive regulating” and is presented with a green arrow. The black curved arrow represents the physiological function and the dotted arrow indicates inhibition.

In addition, we also found FlhF can directly bind to the promoters of *rpoD*, *flgS*, and *fliA* genes respectively (Figure 6). These genes are key regulatory factors during flagellar synthesis. Balaban proposed that FlhF may directly or indirectly influence the FEA-FlgSR pathway to initiate σ54-dependent genes expression in *C. jejuni* (Balaban et al., 2009). We propose FlhF may bind P*flgS* to stimulate *flgS* phosphorylation of *flgR*, cooperating with σ54 factor indirectly, to initiate class II gene transcription synthesis. Meanwhile, FlhF can also directly regulate specific class II gene *flgI*. In addition, FlhF may directly influence the synthesis of class I and III flagellar genes by binding to the *rpoD* and *fliA* promoters respectively. However, the expression of *fliA* is inhibited by FlgM, and interestingly both *fliA* and *flgM* were significantly downregulated in the absence of FlhF (Table 1). Thus, one possible hypothesis is that FlhF directly regulates *fliA* in an independent pathway to promote class III genes synthesis (FlgM being an anti-sigma factor that possibly does not affect *fliA* expression, but its activity). Finally, we proposed a hypothetical model for the regulatory pathway of FlhF in the flagellar system (Figure 7). In addition, in order to further investigate whether there are similar sites between the binding promoters, we compared the putative 26 bp binding sequence in P*flgI* with the P*rpoD*, P*fliA*, and P*flgS* promoters through the MEME website (Bailey, 2002). The MEME analysis identified a similar sequence, an AT-rich region (data not shown). We will explore the conservation of FlhF binding sequences in the future.

**TABLE 1:** Differentially expressed flagellar genes between the *flhF* mutant and wild-type strains.

**Gene_ID**	**Log2FC**	**Class**	**Gene description**
CJJ81176_RS00360	–7.721164828	I	Flagellar biosynthesis protein FlhF
CJJ81176_RS00345	–1.53294197	I	RNA polymerase sigma factor FliA
CJJ81176_RS02660	–1.489019921	I	Flagella export chaperone FliS
CJJ81176_RS00335	–1.108875525	I	Flagellar motor switch protein FliY
CJJ81176_RS00340	–1.055058784	I	Flagellar motor switch protein FliM
CJJ81176_RS00255	–4.034345574	II	Flagellar hook-length control protein FliK
CJJ81176_RS06435	–3.961478247	II	Flagellin B (FlaB)
CJJ81176_RS00265	–3.918053318	II	Flagellar hook protein FlgE
CJJ81176_RS00260	–3.83754024	II	Flagellar basal body rod modification protein
CJJ81176_RS08350	–3.541966567	II	Flagellar hook protein FlgE
CJJ81176_RS07025	–3.424321206	II	Flagellar protein FlgN
CJJ81176_RS07030	–3.19611075	II	Flagellar hook-associated protein FlgK
CJJ81176_RS03320	–3.036794005	II	Flagellar L-ring protein (FlgH)
CJJ81176_RS07020	–2.9346010678	II	Anti-σ factor (FlgM)
CJJ81176_RS07015	–2.87737963	II	Rod assembly protein (FlgJ)
CJJ81176_RS04235	–2.742932491	II	Flagellar hook-associated protein FlgL
CJJ81176_RS07010	–2.710980572	II	Flagellar P-ring protein (FlgI)
CJJ81176_RS00355	–2.073823244	II	MinD/ParA family protein (FlhG)
CJJ81176_RS02555	–1.831517369	II	Flagellar basal body rod protein FlgB
CJJ81176_RS03370	–1.76013941	II	Flagellar hook-basal body protein (FlgG2)
CJJ81176_RS03375	–1.678817425	II	Flagellar basal-body rod protein FlgG
CJJ81176_RS06440	–3.392189439	III	Flagellin A
CJJ81176_RS02655	–1.844789866	III	Flagellar filament capping protein FliD

*Genes with log2 (fold change) > 1.0 or <1.0 with a *p*-value ≤ 0.05 were considered significant. Genes of unknown functions and those that encode hypothetical proteins were not included in this table.*

So far, a number of studies have reported that FlhF is a member of the signal recognition particle (SRP)-related GTPase family regulating the number and position of flagella (Green et al., 2009; Guttenplan et al., 2013). However, no FlhF homologs or functionally similar protein with DNA-binding activity has been reported. In our study, we hypothesis that in addition to being an SPR GTPase, FlhF can also directly influence flagella synthesis by binding to the promoters of flagellar genes in *C. jejuni*. In addition, the GTPase activity of FlhF is not required for flagellar gene transcription in *C. jejuni* (Gulbronson et al., 2016). Therefore, we speculate that the GTPase activity of FlhF may have little to do with the proposed regulation of FlhF here.

In summary, this study demonstrates that FlhF may directly regulate the transcription of flagellar genes by binding to their promoters as a transcriptional regulator. This will help in our attempts to understand the mechanistic role of FlhF in flagellar biosynthetic and bacterial flagellation. We hope this study will be used as foundation for future studies on FlhF function.

## Data Availability Statement

The data is on EBI ENA website with accession number PRJEB34440 (https://www.ebi.ac.uk/ena/data/search?query=PRJEB34440).

## Author Contributions

XL, FR, OG, JH, and XJ conceived and designed the experiments. XL, GC, PH, and QC performed the experiments. XL analyzed the data. XL, JH, and XJ contributed reagents, materials, and analysis tools. XL wrote the manuscript. OG and JH reviewed the manuscript.

## Conflict of Interest

The authors declare that the research was conducted in the absence of any commercial or financial relationships that could be construed as a potential conflict of interest.

## Footnotes

1 http://deweylab.biostat.wisc.edu/rsem/

2 http://www.bioconductor.org/packages/2.12/bioc/html/edgeR.html
