# Supplementary material for: Investigating the Role of FlhF Identifies Novel Interactions With Genes Involved in Flagellar Synthesis in Campylobacter jejuni
Source: Front Microbiol. 2020 Mar 24;11:460. doi: 10.3389/fmicb.2020.00460 (PMC7105676; doi:10.3389/fmicb.2020.00460)
Supplement: Supplementary file 1 [file Data_Sheet_1.docx]

**Table S1. Strains and plasmids used in this study**

| **Strain or plasmid** | | **Description** | **Resistance ^a^** | **Source or reference** |
| --- | --- | --- | --- | --- |
| **Strains** |  |  |  |  |
| *C. jejuni* strains | 81-176 | Wild-type |  | This study |
|  | *flhF*-Kan mutant | *flhF*::kan | Kan | This study |
|  | *flhF* complemented strain | *flhF* mutant containing complementation plasmid (pUOA18-p*metK*- *flhF*) | Kan, Cm | This study |
|  | PAF(Flag) | *flhF* mutant expression of Flag tagged FlhF protein | Kan, Cm | This study |
|  | pMW10 (WT) | Wild-type containing pMW10 | Kan | [^1^] |
|  | p10-P*flgI* (WT) | Wild-type containing pMW10 with P*flgI* | Kan | This study |
|  | p10-P*flgI*-1 (WT) | Wild-type containing pMW10 with P*flgI*-1 | Kan | This study |
|  | p10-P*flgI*-2 (WT) | Wild-type containing pMW10 with P*flgI*-2 | Kan | This study |
|  | p10-P*flgI*-3 (WT) | Wild-type containing pMW10 with P*flgI*-3 | Kan | This study |
|  | p10-P*flgI*-4 (WT) | Wild-type containing pMW10 with P*flgI*-4 | Kan | This study |
|  | p10-P*flgI*-5 (WT) | Wild-type containing pMW10 with P*flgI*-5 | Kan | This study |
|  | pMW10 (*flhF*-Kan) | *flhF*-Kan containing pMW10 | Kan | [^1^] |
|  | p10-P*flgI* (*flhF*-Kan) | *flhF*-Kan containing pMW10 with P*flgI* | Kan | This study |
|  | p10-P*flgI*-1 (*flhF*-Kan) | *flhF*-Kan containing pMW10 with P*flgI*-1 | Kan | This study |
|  | p10-P*flgI*-2 (*flhF*-Kan) | *flhF*-Kan containing pMW10 with P*flgI*-2 | Kan | This study |
|  | p10-P*flgI*-3 (*flhF*-Kan) | *flhF*-Kan containing pMW10 with P*flgI*-3 | Kan | This study |
|  | p10-P*flgI*-4 (*flhF*-Kan) | *flhF*-Kan containing pMW10 with P*flgI*-4 | Kan | This study |
|  | p10-P*flgI*-5 (*flhF*-Kan) | *flhF*-Kan containing pMW10 with P*flgI*-5 | Kan | This study |
| *E.coli* strains | DH5α | Cloning host |  | TaKaRa |
|  | BL21(DE3) | T7 expression host |  |  |
| **Plasmids** |  |  |  |  |
|  | pMD-19T(simple) | Cloning and suicide vector | Amp | TaKaRa |
|  | pRK2013 | Helper plasmid | Kan | Biomedal |
|  | pUOA18 | Shuttle vector | Cm | [^2^] |
|  | pRY107 | PCR templet for Kan^r^ cassette | Kan | [^2^] |
|  | pCold I | Expression vector | Amp | This study |
|  | pET-30a | Expression vector | Kan | This study |
|  | pMW10 | Promoter shuttle vector containing promoterless lacZ | Kan | [^1^] |
|  | pMD19T-*flhF*-upstream *-kan*^r^-downstream | Suicide plasmid for *flhF* mutant construction | Amp, Kan | This study |
|  | pUOA18-p*metK*- *flhF* | Complementation plasmid | Cm | This study |
|  | pUOA18-p*metK*-FLAG- *flhF* (PAF) | Complementation plasmid with FLAG-tag | Cm | This study |
|  | pET30a-*flhF* | Expression vector for expressing FlhF protein | Kan | This study |
|  | pCold I-*cmeR* | Expression vector for expressing CmeR protein | Amp | This study |
|  | pMW10-P*flgI* | pMW10 with P*flgI* | Kan | This study |
|  | pMW10-P*flgI*-1 | pMW10 with P*flgI*-1 | Kan | This study |
|  | pMW10-P*flgI*-2 | pMW10 with P*flgI*-2 | Kan | This study |
|  | pMW10-P*flgI*-3 | pMW10 with P*flgI*-3 | Kan | This study |
|  | pMW10-P*flgI*-4 | pMW10 with P*flgI*-4 | Kan | This study |
|  | pMW10-P*flgI*-5 | pMW10 with P*flgI*-5 | Kan | This study |

^a^ Abbreviations: Kan, kanamycin; Cm, Chloramphenicol; Amp, ampicillin.

**Reference**

1. Wosten MM, Boeve M, Koot MG, van Nuenen AC, van der Zeijst BA. 1998. Identification of *Campylobacter jejuni* promoter sequences. J Bacteriol 180:594–599.

2. Ren F, Lei T, Song Z, Yu T, Li Q, Huang J, Jiao X. 2018. Could FlhF be a key element that controls *Campylobacter jejuni* flagella biosynthesis in the initial assembly stage? Microbiol Res 207:240–248.

**Table S2. Primers used in this study**

| **Primers** | | **Sequence (5’-3’)** | | **Target gene/fragment** | |  |
| --- | --- | --- | --- | --- | --- | --- |
| For strain construction | |  | |  | |  |
|  | pMD19T-upstream-F | TTTGCACGCCTGCCGTTCGACGATT*GTCGAC*CATTATCAAGCACTTTCCGTT (*Sal* I) | | The upstream fragment of *flhF* | |  |
|  | pMD19T-upstream-R | TGTAAGTTCTTTAGTGATTGTCTTAGCATTATCCTGCAGCCAAGCAAATAAACTTCGCA | |  |  |  |
|  | pMD19T*-kan^r^-*F | CCATTTTGACTCATGAGATTGCGAAGTTTATTTGCTTGGCTGCAGGATAATGCTAAGAC | | Kanamycin resistance cassette | |  |
|  | pMD19T*-kan^r^-*R | GAGTGTAATTTTGCCTTTAGGCATGATATAAGGAATATAACTGCAGCGCTTATCAATAT | |  |  |  |
|  | pMD19T-downstream-F | TGCTTTGCCCATTCTATAGATATATTGATAAGCGCTGCAGTTATATTCCTTATATCATGCCTAAAG | | The downstream fragment of *flhF* | |  |
|  | pMD19T-downstream-R | CGGTACGCGCGGATCTTCCAGAGAT*GAGCTC*CAGCAGGCTATACTCATACTTCAG (*Sac* I) | |  |  |  |
|  | Flag-*flhF-*F | GT*GGATCC*ATGGATTACAAGGATGACGACGATAAGATGGGACAACTTATACAT (*B*amHI) | | *flhF* with Flag tag | |  |
|  | Flag-*flhF-*R | GCGC*GAGCTC*TTATTCATTATTTTTTCC (*Sac* I) | |  |  |  |
|  | pMW10-P*flgI*-F | TCAAACATGAGAATT*CCCGGG*AGAAAATTTAGATTTAAAGACT (*Sma* I) | | The promoter of *flgI* | |  |
|  | pMW10-P*flgI*-R | CTCTAGCTAGAAGCT*TCTAGA*TGGATTCTTCCTTTAATCT (*Xba* I) | |  |  |  |
|  | pMW10-P*flgI*-1-F | TCAAACATGAGAATT*CCCGGG*AATTTAATCATTTTTGAACACCACT (*Sma* I) | | A fragment of P*flgI* | |  |
|  | pMW10-P*flgI*-1-R | CTCTAGCTAGAAGCT*TCTAGA*TGGATTCTTCCTTTAATCTATTGTT (*Xba* I) | |  |  |  |
|  | pMW10-P*flgI*-2-F | TCAAACATGAGAATT*CCCGGG*CAACCATAAAAACTCCCGAAA (*Sma* I) | | A fragment of P*flgI* | |  |
|  | pMW10-P*flgI*-2-R | CTCTAGCTAGAAGCT*TCTAGA*TGGATTCTTCCTTTAATCTATTGTT (*Xba* I) | |  |  |  |
|  | pMW10-P*flgI*-3-F | TCAAACATGAGAATT*CCCGGG*CCAAAATTTTCAAAAAGTCAAAG (*Sma* I) | | A fragment of P*flgI* | |  |
|  | pMW10-P*flgI*-3-R | CTCTAGCTAGAAGCT*TCTAGA*TGGATTCTTCCTTTAATCTATTGTT (*Xba* I) | |  |  |  |
|  | pMW10-P*flgI*-4-F | TCAAACATGAGAATT*CCCGGG*AAGAAATTTGGATCAACTAGCTTAA (*Sma* I) | | A fragment of P*flgI* | |  |
|  | pMW10-P*flgI*-4-R | CTCTAGCTAGAAGCT*TCTAGA*TGGATTCTTCCTTTAATCTATTGTT (*Xba* I) | |  |  |  |
|  | pMW10-P*flgI*-5-F | TCAAACATGAGAATT*CCCGGG*GCTTTTACGCTTCTTAACTTACTTG (*Sma* I) | | A fragment of P*flgI* | |  |
|  | pMW10-P*flgI*-5-R | CTCTAGCTAGAAGCT*TCTAGA*TGGATTCTTCCTTTAATCTATTGTT (*Xba* I) | |  |  |  |
| For ChIP-qPCR | |  | |  | |  |
| P*flgI* RT-F | | AACACCACTCAACCATA | | A fragment of P*flgI* | |  |
| P*flgI* RT-R | | CTTTAATCTATTGTTGATGA | |  |  |  |
| P*flaB* RT-F | | TTCTTTTAAGAATGCTG | | A fragment of P*flaB* | |  |
| P*flaB* RT-R | | CCATTTTAAATCCTTTC | |  |  |  |
| For qRT-PCR | |  | |  | |  |
|  | *fliS* RT-F | CAAGAGCTAAAGTAGCCATA | | A fragment of *fliS* | |  |
|  | *fliS* RT-R | CCCGCTTAAATAATGTG | |  |  |  |
|  | *fliM* RT-F | CAAGCGGAATGGTAAAT | | A fragment of *fliM* | |  |
|  | *fliM* RT-R | CACTTCAGCACGACCGA | |  |  |  |
|  | *flaB* RT-F | GTGCAATGGCGGTTATG | | A fragment of *flaB* | |  |
|  | *flaB* RT-R | GTTGATTCGGCTGCTTT | |  |  |  |
|  | *flgE* RT-F | AAGCGGACTACAAGCACATC | | A fragment of *flgE* | |  |
|  | *flgE* RT-R | AACTTACTGAAACGCCAAGA | |  |  |  |
|  | *flgH* RT-F | GGAGGTGCTTTAACAGGAAG | | A fragment of *flgH* | |  |
|  | *flgH* RT-R | CCGATGTCATAAGGACGAAT | |  |  |  |
|  | *flgI* RT-F | TGGTTACAGCCAAACTTCCG | | A fragment of *flgI* | |  |
|  | *flgI* RT-R | CATTAGCTCCGCCCATTACG | |  |  |  |
|  | *flaA* RT-F | GAACAGGACTTGGAGCTTTG | | A fragment of *flaA* | |  |
|  | *FlaA* RT-R | CGTTACCATCTCCGTCTTTG | |  |  |  |
|  | For EMSA | |  | |  | |
|  | pET30a-FlhF-F | GCCATGGCTGATATCGGATCCATGGGACAACTTATACAT (*B*amHI) | | *flhF* gene | |  |
|  | pET30a-FlhF-R | GTGGTGGTGGTGGTGCTCGAGTTATTCATTATTTTTTCC (*Xho* I) | |  |  |  |
|  | P*flgI*-F | TGCCTGCAGGTCGACGATAGAAAATTTAGATTTAAAGACT | | The promoter of *flgI* | |  |
|  | P*flgI*-R | TGGATTCTTCCTTTAATCT | |  |  |  |
|  | P*flgI*-1-F | TGCCTGCAGGTCGACGATAATTTAATCATTTTTGAACACCACT | | A fragment of the *flgI* promoter | |  |
|  | P*flgI*-1-R | TGGATTCTTCCTTTAATCTATTGTT | |  |  |  |
|  | P*flgI*-2-F | TGCCTGCAGGTCGACGATCAACCATAAAAACTCCCGAAA | | A fragment of the *flgI* promoter | |  |
|  | P*flgI*-2-R | TGGATTCTTCCTTTAATCTATTGTT | |  |  |  |
|  | P*flgI*-3-F | TGCCTGCAGGTCGACGATCCAAAATTTTCAAAAAGTCAAAG | | A fragment of the *flgI* promoter | |  |
|  | P*flgI*-3-R | TGGATTCTTCCTTTAATCTATTGTT | |  |  |  |
|  | P*flgI*-4-F | TGCCTGCAGGTCGACGATAAGAAATTTGGATCAACTAGCTTAA | | A fragment of the *flgI* promoter | |  |
|  | P*flgI*-4-R | TGGATTCTTCCTTTAATCTATTGTT | |  |  |  |
|  | P*flgI*-5-F | TGCCTGCAGGTCGACGATGCTTTTACGCTTCTTAACTTACTTG | | A fragment of the *flgI* promoter | |  |
|  | P*flgI*-5-R | TGGATTCTTCCTTTAATCTATTGTT | |  |  |  |
|  | P*flgI*-6-F | TGCCTGCAGGTCGACGATAGAAAATTTAGATTTAAAGACTTTA | | A fragment of the *flgI* promoter | |  |
|  | P*flgI*-6-R | TTCTTTGACTTTTTGAAAATT | |  |  |  |
|  | P*fliK*-F | TGCCTGCAGGTCGACGATAATTGGAACACTTATTGCTTAA | | The promoter of *fliK* | |  |
|  | P*fliK*-R | AAGCTCTATCAAAAGAAGTTTTA | |  |  |  |
|  | P*flaB*-F | TGCCTGCAGGTCGACGATTACAATAGTTAAATCTTAAAATCAC | | The promoter of *flaB* | |  |
|  | P*flaB*-R | CTTTTAAATCCTTTCAAAATATT | |  |  |  |
|  | P*flgE*-F | TGCCTGCAGGTCGACGATGGGTGTGATTTTGGTAATT | | The promoter of *flgE* | |  |
|  | P*flgE*-R | TCTTAAATCCTTTATAAAATATTG | |  |  |  |
|  | P*flaA*-F | TGCCTGCAGGTCGACGATAATTGAAGATGAAAGAGAGTATT | | The promoter of *flaA* | |  |
|  | P*flaA*-R | TTTAAATCCTTTTAAATAATTTC | |  |  |  |
|  | P*flgL*-F | TGCCTGCAGGTCGACGATACCAGTTTCACCACTCGG | | The promoter of *flgL* | |  |
|  | P*flgL*-R | TTTCCTCTAAAGTATTAAAGTTAAA | |  |  |  |
|  | P*rpoD*-F | TGCCTGCAGGTCGACGATAAATATCGACCTAAAGCGT | | The promoter of *rpoD* | |  |
|  | Pr*poD*-R | TTGATTATCCTTGAAATTTATAT | |  |  |  |
|  | P*rpoN*-F | TGCCTGCAGGTCGACGATATCCTTTGTTAAAATAGATTTG | | The promoter of *rpoN* | |  |
|  | Pr*poN*-R | ATAACGCCATTAATCCCC | |  |  |  |
|  | P*fliA*-F | TGCCTGCAGGTCGACGATTGCAGATGCAAACATTAA | | The promoter of *fliA* | |  |
|  | P*fliA*-R | TATCAAGCACTTTCCGTT | |  |  |  |
|  | P*flgS*-F | TGCCTGCAGGTCGACGATAAAATAAAAAAATTTGTAAAAA | | The promoter of *flgS* | |  |
|  | P*flgS*-R | TCATTTATCATTCCTCTAATAAA | |  |  |  |
|  | P*flgR*-F | TGCCTGCAGGTCGACGATGGAGCTAGAATCAGTGTTAAG | | The promoter of *flgR* | |  |
|  | P*flgR*-R | AATTCATCCTAAGCCTTTTA | |  |  |  |
|  | pcold-CmeR-F | ATGGAGCTCGGTACCCTCGAGATGAACTCAAATAGAAC (*Xho* I) | | *cmeR* gene | |  |
|  | pcold-CmeR-F | CTATCTAGACTGCAGGTCGACTTAAGCTTTGGAGCTATTG (*Sal* I) | |  |  |  |
|  | P*cmeA*-F | TGCCTGCAGGTCGACGATCTAAATGGAATCAATAGCTCC | | The promoter of *cmeA* | |  |
|  | P*cmeA*-R | GCACAACACCTAAAGCTAAAA | |  |  |  |
|  | Mutant 1-F | AATAAATTTGGATCAACTAGCTTAAG | | The fragment of Point mutation 1 | |  |
|  | Mutant 1-R | CTTAAGCTAGTTGATCCAAATTTATT | |  |  |  |
|  | Mutant 2-F | AAGAAATGTGGATCAACTAGCTTAAG | | The fragment of Point mutation 2 | |  |
|  | Mutant 2-R | CTTAAGCTAGTTGATCCACATTTCTT | |  |  |  |
|  | Mutant 3-F | AAGAAATTTGGAGCAACTAGCTTAAG | | The fragment of Point mutation 3 | |  |
|  | Mutant 3-R | CTTAAGCTAGTTGCTCCAAATTTCTT | |  |  |  |
|  | Mutant 4-F | AAGAAATTTGGATCAACGAGCTTAAG | | The fragment of Point mutation 4 | |  |
|  | Mutant 4-R | CTTAAGCTCGTTGATCCAAATTTCTT | |  |  |  |
|  | Mutant 5-F | AAGAAATTTGGATCAACTAGCTTGAG | | The fragment of Point mutation 5 | |  |
|  | Mutant 5-R | CTCAAGCTAGTTGATCCAAATTTCTT | |  |  |  |
|  | 26bp-binding site-F | AAGAAATTTGGATCAACTAGCTTAAG | | The fragment of FlhF-binding site | |  |
|  | 26bp-binding site-R | AAGAAATTTGGATCAACTAGCTTAAG | |  |  |  |
|  | For transcriptional fusion | |  | |  | |
|  | LacZ-F | ATGAAAGGGAATTCACTGG | | LacZ gene | |  |
|  | LacZ-R | TTATTTTTGACACCAGACCAA | |  |  |  |
|  | Kan^r^-F | GATAATGCTAAGACAATCACTAAAGAACTT | | Kanamycin resistance cassette | |  |
|  | Kan^r^-R | CGCTTATCAATATATCTATAGAATGGGC | |  |  |  |

**Table S3.** Differentially expressed genes between the *flhF* mutant and wild-type strains. Genes with log2 (fold change)>1.0 or <1.0 with a p-value ≤0 .05 were considered significant.

| Gene_ID | Log2FC | Pvalue | Regulate |
| --- | --- | --- | --- |
| CJJ81176_RS00360  CJJ81176_RS08605  CJJ81176_RS07955  CJJ81176_RS08415  CJJ81176_RS08370  CJJ81176_RS08455  CJJ81176_RS04135  CJJ81176_RS00255  CJJ81176_RS04735  CJJ81176_RS06435  CJJ81176_RS00265  CJJ81176_RS00260  CJJ81176_RS08480  CJJ81176_RS08505  CJJ81176_RS08610  CJJ81176_RS08350  CJJ81176_RS07025  CJJ81176_RS06440  CJJ81176_RS00275  sRNA0071  CJJ81176_RS07030  CJJ81176_RS03320  CJJ81176_RS00350  CJJ81176_RS06045  CJJ81176_RS07020  CJJ81176_RS08530  CJJ81176_RS07015  CJJ81176_RS08565  CJJ81176_RS01175  CJJ81176_RS04235  CJJ81176_RS01885  CJJ81176_RS07010  CJJ81176_RS08555  sRNA0072  CJJ81176_RS02650  CJJ81176_RS08485  CJJ81176_RS03930  CJJ81176_RS08490  CJJ81176_RS08500  sRNA0073  CJJ81176_RS08535  CJJ81176_RS08405  CJJ81176_RS00355  CJJ81176_RS08450  CJJ81176_RS07585  CJJ81176_RS08495  CJJ81176_RS00645  CJJ81176_RS08615  CJJ81176_RS02655  CJJ81176_RS02555  CJJ81176_RS02665  CJJ81176_RS03370  CJJ81176_RS07830  CJJ81176_RS08520  CJJ81176_RS07925  CJJ81176_RS00270  CJJ81176_RS03375  CJJ81176_RS01400  CJJ81176_RS00345  CJJ81176_RS02660  CJJ81176_RS06950  CJJ81176_RS04980  CJJ81176_RS02055  CJJ81176_RS00335  CJJ81176_RS00340  CJJ81176_RS04985  CJJ81176_RS06400  CJJ81176_RS07945  CJJ81176_RS02795  CJJ81176_RS03760  CJJ81176_RS03765  CJJ81176_RS01980  CJJ81176_RS04480  CJJ81176_RS03475  CJJ81176_RS07140  CJJ81176_RS03380  CJJ81176_RS05985  CJJ81176_RS02005  CJJ81176_RS06540  CJJ81176_RS02140  CJJ81176_RS02680  CJJ81176_RS06545  CJJ81176_RS02010  CJJ81176_RS03635  CJJ81176_RS03630  CJJ81176_RS00035  CJJ81176_RS04165  CJJ81176_RS02135  CJJ81176_RS05930  CJJ81176_RS00175  CJJ81176_RS00190  CJJ81176_RS04170  CJJ81176_RS02130  CJJ81176_RS00145  sRNA0068  CJJ81176_RS05935  sRNA0049  CJJ81176_RS00180  CJJ81176_RS02475 | -7.721164828  -7.115839186  -4.916167885  -4.82991478  -4.74738335  -4.595421604  -4.362586221  -4.034345574  -4.014527991  -3.961478247  -3.918053318  -3.83754024  -3.718705052  -3.681310825  -3.551551697  -3.541966567  -3.424321206  -3.392189439  -3.382023672  -3.290292143  -3.19611075  -3.036794005  -2.98712032  -2.973976481  -2.934601068  -2.917769415  -2.87737963  -2.819082148  -2.818189753  -2.742932491  -2.711310563  -2.710980572  -2.635824705  -2.52097114  -2.460705885  -2.434181994  -2.406432563  -2.332455697  -2.264480066  -2.245037186  -2.186003855  -2.087401704  -2.073823244  -1.921463931  -1.908330254  -1.887687096  -1.869106649  -1.853080747  -1.844789866  -1.831517369  -1.810674941  -1.76013941  -1.743113855  -1.692989533  -1.691055791  -1.686690943  -1.678817425  -1.547718558  -1.53294197  -1.489019921  -1.40473596  -1.379291104  -1.191899266  -1.108875525  -1.055058784  -0.892020189  -0.87324746  0.830467655  0.84383003  0.900872062  1.019211504  1.050930582  1.080833516  1.082759415  1.153837256  1.180086951  1.192324348  1.197802332  1.214945035  1.233001829  1.253841476  1.270350472  1.289299019  1.348248289  1.474526021  1.482166534  1.491696226  1.533721243  1.561042585  1.621285454  1.659947952  1.670336867  1.70231057  1.764035077  1.812738091  1.91122736  2.132144673  2.138367441  2.160401707 | 4.73376E-82  3.16824E-66  1.21301E-54  7.83859E-25  1.72543E-24  6.89764E-23  1.60493E-33  7.77722E-39  2.58302E-40  4.5639E-55  4.05237E-49  1.15356E-28  1.61008E-14  2.86668E-14  3.07409E-13  4.01987E-46  1.21156E-27  1.82857E-38  3.27502E-18  1.2159E-23  2.13995E-30  3.96427E-25  6.38531E-12  3.38684E-19  3.33066E-16  2.55281E-09  4.44673E-12  8.31998E-09  1.10102E-09  9.81537E-25  6.614E-19  6.49831E-20  6.54436E-08  1.68944E-08  5.61421E-16  5.32287E-07  1.52401E-11  1.43088E-06  2.83052E-06  3.15132E-06  5.55684E-06  1.30622E-05  7.99669E-13  4.68434E-05  6.17964E-05  6.03815E-05  2.26326E-10  8.29487E-05  4.33491E-13  4.18525E-10  9.99284E-05  3.26199E-10  1.99527E-06  0.000259356  5.67725E-07  0.000435906  3.45351E-07  0.001457554  2.63986E-07  1.45205E-06  1.5288E-05  2.91964E-05  0.000928575  0.000382372  0.001666766  0.001481879  0.002127408  0.002664635  0.002043976  0.001222854  0.000383069  0.001176577  0.001345731  0.001222057  0.000828492  0.000836441  0.001311394  0.001329555  1.58266E-05  1.60662E-06  0.000280669  2.04883E-05  0.000976274  0.001933851  0.002518372  1.78066E-05  2.20429E-05  1.47125E-07  0.000272873  1.66666E-06  1.31336E-05  1.9367E-06  1.47901E-07  7.12217E-08  1.08122E-05  4.58902E-06  5.95583E-06  1.12562E-06  2.72339E-06 | down  down  down  down  down  down  down  down  down  down  down  down  down  down  down  down  down  down  down  down  down  down  down  down  down  down  down  down  down  down  down  down  down  down  down  down  down  down  down  down  down  down  down  down  down  down  down  down  down  down  down  down  down  down  down  down  down  down  down  down  down  down  down  down  down  down  down  up  up  up  up  up  up  up  up  up  up  up  up  up  up  up  up  up  up  up  up  up  up  up  up  up  up  up  up  up  up  up  up |


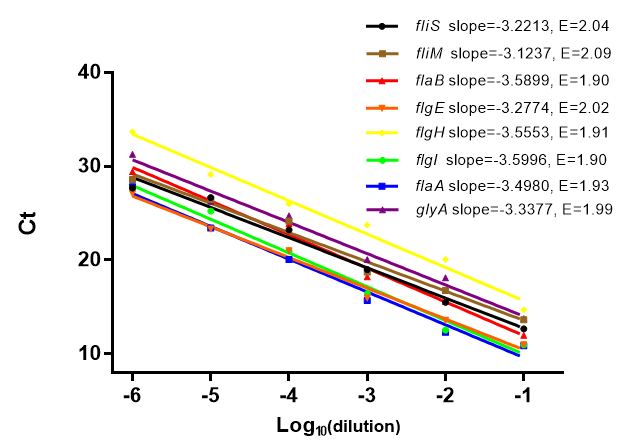


**Fig. S1. Standard curve analysis of qRT-PCR amplification efficiencies**

A series of 10-fold diluted cDNA were used as templates and the standard curves were generated for *fliS*, *fliM*, *flaB*, *flgE*, *flgH*, *flgI*, *flaA* genes and the reference gene *glyA*. The Log_10_ (dilution of cDNA) is shown on the horizontal axis and the cycle threshold (CT) values on the vertical axis. R^2^≥0.98, The PCR efficiency (E) was calculated using the formula “E=10^(-1/-slope)^”. Results showed that the amplification efficiency of each pair of primers were close to 2 exhibiting the strong linear trend.

**Table S4. The cycle threshold (CT) values of the reference gene *glyA* in qRT-PCR.**

| Biological repeatability | CT (WT) | CT (*flhF* mutant) |
| --- | --- | --- |
| 1 | 19.40115 | 19.77988 |
| 2 | 19.29575 | 19.31345 |
| 3 | 18.92189 | 18.80881 |


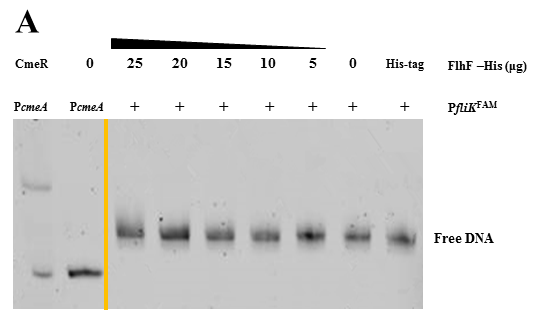

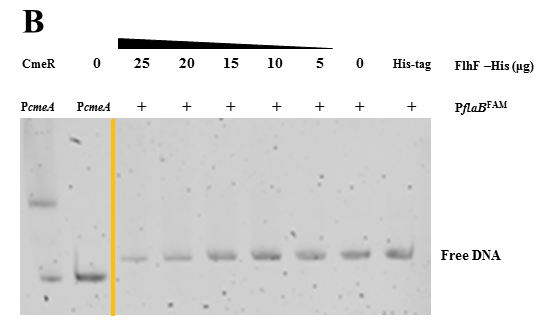


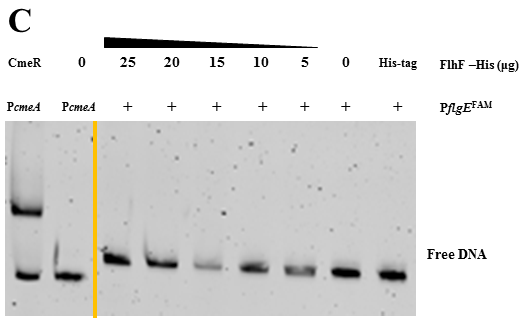

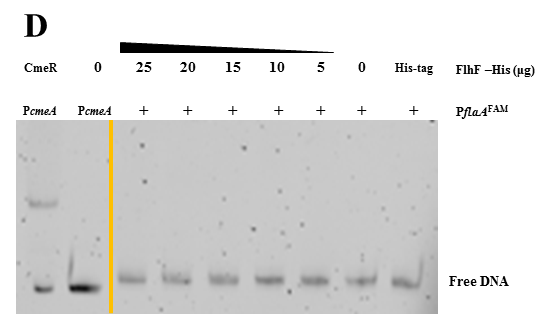


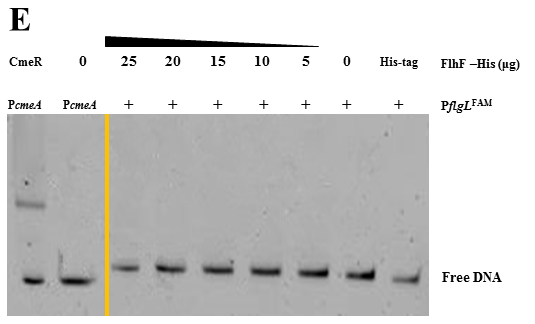


**Fig. S2. EMSA analysis of FlhF with the promoters of *fliK, flaB, flgE, flaA, flgL*.**

The fluorescently labeled DNA probes of the gene promoter were incubated with purified FlhF-6His at different concentrations. (A) The promoter of *fliK*; (B) *flaB*; (C) *flgE*; (D) *flaA*; (E) *flgL*. Each reaction was verified to be specific by adding 10-fold non-specific competitor (Poly(dI:dC)). For a negative control, synthesized His-tag was incubated with flgI promoter, denoted as negative control (NC). For positive controls, cmeA promoter was incubated with the purified CmeR protein, and cmeA promoter alone, denoted as positive control (PC). The “+” symbol indicates the presence of FlhF-6His. Results showed that FlhF did not bind to their promoters.


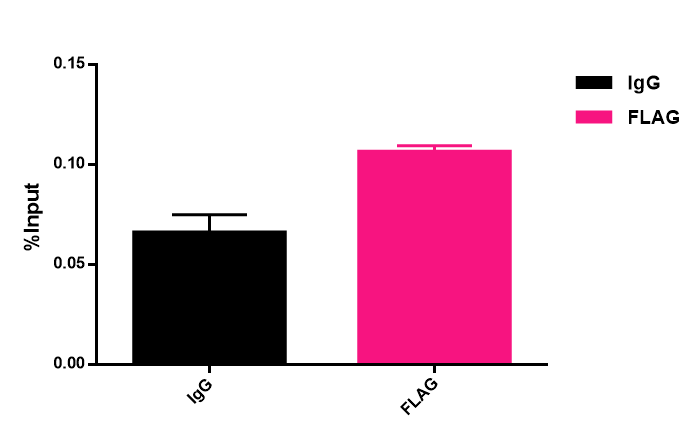


**Fig. S3. Fold enrichment of the *flaB* promoter in ChIP samples, as measured via ChIP-qPCR.**

Genome fragments isolated from wild-type and overexpression strains were immunoprecipitated with corresponding antibodies, and analyzed by real-time PCR using primer sets corresponding to transcriptional start site regions of the *flaB*. For ChIP-qPCR experiments, untreated chromatin was decrosslinked and purified by boiling for use as an "input" control. The relative enrichment of the candidate gene promoter was performed by qRT-PCR and represents the value of immunoprecipitation. These values were normalized to values obtained using each promoter of the unlabeled wild type precipitate to account for non-specific enrichment. Results represent mean enrichment as measured by qPCR in at least three biological replicate experiments. Data are presented as mean + SD. Data were analysed by one-sample t-test (*P< 0.05, **P< 0.01, ***P< 0.001) to estimate the significance of fold change between FlhF-ChIP samples and IgG control samples. The results show the promoter of *flaB* was not enriched in the FlhF-ChIP samples.


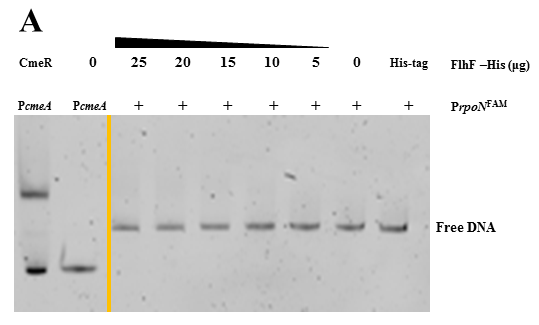

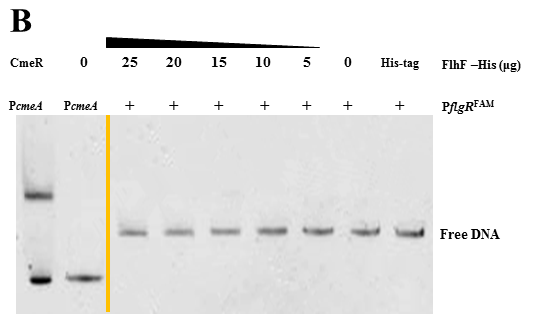


**Fig. S4. EMSA analysis of FlhF with the promoters of *rpoN*, *flgR*.**

The fluorescently labeled DNA probes of the gene promoter were incubated with purified FlhF-6His at different concentrations. (A) The promoter of *rpoN*; (B) *flgR*. For a negative control, synthesized His-tag was incubated with flgI promoter, denoted as negative control (NC). Each reaction was verified to be specific by adding 10-fold non-specific competitor (Poly(dI:dC)). For positive controls, cmeA promoter was incubated with the purified CmeR protein, and cmeA promoter alone, denoted as positive control (PC). The “+” symbol indicates the presence of FlhF-6His. Results showed that FlhF did not bind to the promoters of *rpoN* and *flgR*.


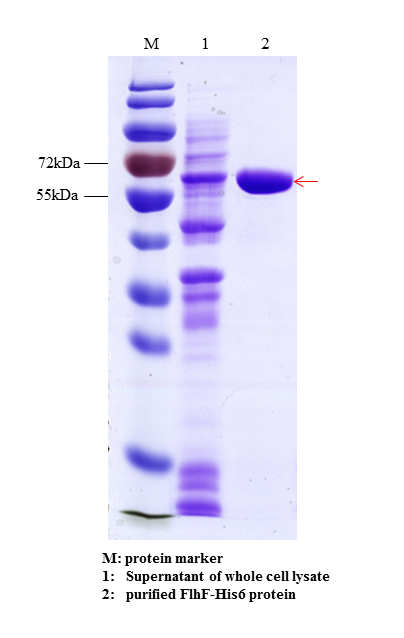


**Fig. S5. SDS-PAGE analysis of induced expression and purification of FlhF-His6 protein.**

FlhF-His6 protein were expressed in *E. coli* DE3 system containing pET-30-FlhF by using the His Bind Purification Kit. M: marker. Lane 1: Supernatants of whole cell lysate. Lane2: purification of FlhF-His6 protein by using the His Bind Purification Kit. The arrowhead indicates the 61.35-kDa FlhF-His6 protein. Data showed the purity of the FlhF preparations was well.
